# Hypophagia and body weight loss by tirzepatide are accompanied by fewer GI adverse events compared to semaglutide in preclinical models

**DOI:** 10.1126/sciadv.adu1589

**Published:** 2025-06-18

**Authors:** Tito Borner, Allison M. Pataro, Sarah A. Doebley, Charles D. Furst, Alex D. White, Serena X. Gao, Angela Chow, Marcos J. Sanchez-Navarro, Misgana Y. Ghidewon, Julia G. Halas, Allaha Z. Mohiby, Francis S. Willard, Harvey J. Grill, Minrong Ai, Ricardo J. Samms, Matthew R. Hayes, Bart C. De Jonghe

**Affiliations:** ^1^ Department of Biobehavioral Health Sciences, University of Pennsylvania, School of Nursing, Philadelphia, PA, USA.; ^2^ Department of Psychiatry, University of Pennsylvania, Perelman School of Medicine, Philadelphia, PA, USA.; ^3^ Department of Biological Sciences, University of Southern California, Los Angeles, CA, USA.; ^4^ Lilly Research Laboratories, Eli Lilly and Company, Indianapolis, IA, USA.; ^5^ Institute of Diabetes, Obesity and Metabolism and School of Arts and Sciences, University of Pennsylvania, Philadelphia, PA, USA.

## Abstract

Glucagon-like peptide-1 receptor (GLP-1R)/glucose-dependent insulinotropic peptide receptor (GIPR) agonistic analogs have yielded superior results in enhancing glycemic control and weight management compared to GLP-1R agonism alone. Intriguingly, GIPR agonism appears to induce antiemetic effects, potentially alleviating part of the nausea and vomiting side effects common to GLP-1R agonists like semaglutide. Here, we show in rats and shrews that GIPR agonism blocks emesis and attenuates other malaise behaviors elicited by GLP-1R activation while maintaining reduced food intake and body weight loss and improved glucose tolerance. The GLP-1R/GIPR agonist tirzepatide induced significantly fewer side effects than equipotent doses of semaglutide. These findings underscore the therapeutic potential of combined pharmaceutical strategies activating both incretin systems, leading to enhanced therapeutic index and reduced occurrence of nausea and vomiting for obesity and diabetes treatments.

## Teaser

Tirzepatide exhibits a greater therapeutic index compared to semaglutide in preclinical models of nausea and emesis.

## INTRODUCTION

Long-acting agonists targeting the glucagon-like peptide-1 receptor (GLP-1R) are the most efficacious treatment to combat the obesity epidemic [see (*1*–*3*) for review]. Semaglutide is the first Food and Drug Administration (FDA)–approved medication to show double-digit weight loss in people with overweight/obesity without type 2 diabetes (*4*). Despite the widespread adoption and treatment success of semaglutide, the drug has a similar profile of adverse events to previous generations of GLP-1–based therapeutics in causing nausea and vomiting in patients. Strategies to reduce side effects, such as slow dose escalation, unfortunately do not fully mitigate nausea, vomiting, and gastrointestinal (GI) distress, which are the primary reasons for treatment discontinuation (*5*). For example, in a recent clinical trial, 44.2% of patients receiving semaglutide reported nausea and 24.8% reported vomiting (*4*). Pooled data from the STEP 1-3 trials indicate that these side effects mostly occurred within the first 20 weeks of treatment and were mild to moderate in severity; however, they led to dose reduction or temporary treatment interruption in ~12.5% of the participants and permanent treatment discontinuation in ~4.3% of subjects (*6*). Therefore, reducing the incidence of adverse GI events from GLP-1R agonist therapeutics may improve treatment adherence and quality of life. To this end, exploring how GLP-1R agonists may be pharmaceutically combined with additional targets, such as glucose-dependent insulinotropic peptide (GIP) analogs, may achieve these goals.

GIP is a gut hormone historically characterized as an incretin hormone for its role in regulating postprandial plasma glucose concentrations by stimulating insulin secretion (*3*, *7*, *8*). In contrast to GLP-1, which is used with success for the treatment of type 2 diabetes (T2DM) and obesity, GIP receptor (GIPR)–based agonism alone produces limited biological and pharmacological effects (*9*, *10*). Despite this, renewed interest in GIP-based pharmacology has emerged via enhanced metabolic efficacy in several preclinical studies and clinical trials assessing combined co-agonism of the GIPR and GLP-1R (*11*–*15*).

Positive clinical results from tirzepatide (originally named LY3298176) (*15*–*17*), a long-acting “sequence-mixed” GIP and GLP-1 receptor agonist, led to FDA approval for T2DM and obesity treatment. Early T2DM trials showed tirzepatide improved clinical efficacy beyond GLP-1R agonist alone (*12*, *15*). Phase 3 clinical trials in people with T2DM (SURPASS) and obesity (SURMOUNT) (*16*–*21*) support early findings and demonstrate advantages of tirzepatide in glycemia and weight management compared to GLP-1R monotherapies. These studies highlighted that tirzepatide led to greater reductions in body weight loss in a higher percentage of patients compared to semaglutide treatment and showed tirzepatide had superior glucoregulatory effects, reflected by greater reductions in HbA1c levels. Notably, a recent review comparing the effects of semaglutide and tirzepatide, derived from STEP 2, SURPASS-1, and SURPASS-2 trials, highlights potential differences in nausea and emesis comparing tirzepatide and semaglutide, suggesting that not only does tirzepatide lead to greater metabolic improvements, but it also appears to be better tolerated by patients in these clinical trials (*22*).

Notably, the lack of standardized protocols for evaluating nausea and emesis adverse events, coupled with the inherent challenge of accurately assessing subjective feelings of nausea in humans, and variations in dose-escalation protocols and participant selection criteria, renders drawing definitive conclusions about the illness behaviors elicited by these therapeutics difficult. In addition, one could argue that some of these effects may be due to the unique intrinsic properties of tirzepatide versus semaglutide (e.g., pharmacodynamic and pharmacokinetic profiles, receptor pharmacology, biodistribution, and the distribution of nuclei functionally targeted from brain penetrance) (*23*, *24*). Therefore, the use of expanded preclinical models to study malaise is critical to resolve these clinical distinctions and discrepancies while simultaneously deepening our understanding of the GIP and GLP-1 systems and their pharmacological benefits when coactivated. Here, using a multispecies approach (mice, rats, and shrews), we investigated the effects on glucose homeostasis, food intake, body weight, and several malaise behaviors of multiple doses and combinations of GLP-1R, GIPR, and GLP-1R/GIPR agonists.

## RESULTS

### GIPR agonism potentiates semaglutide-induced anorexia and body weight loss in diet-induced obese mice

**Fig. 1.**
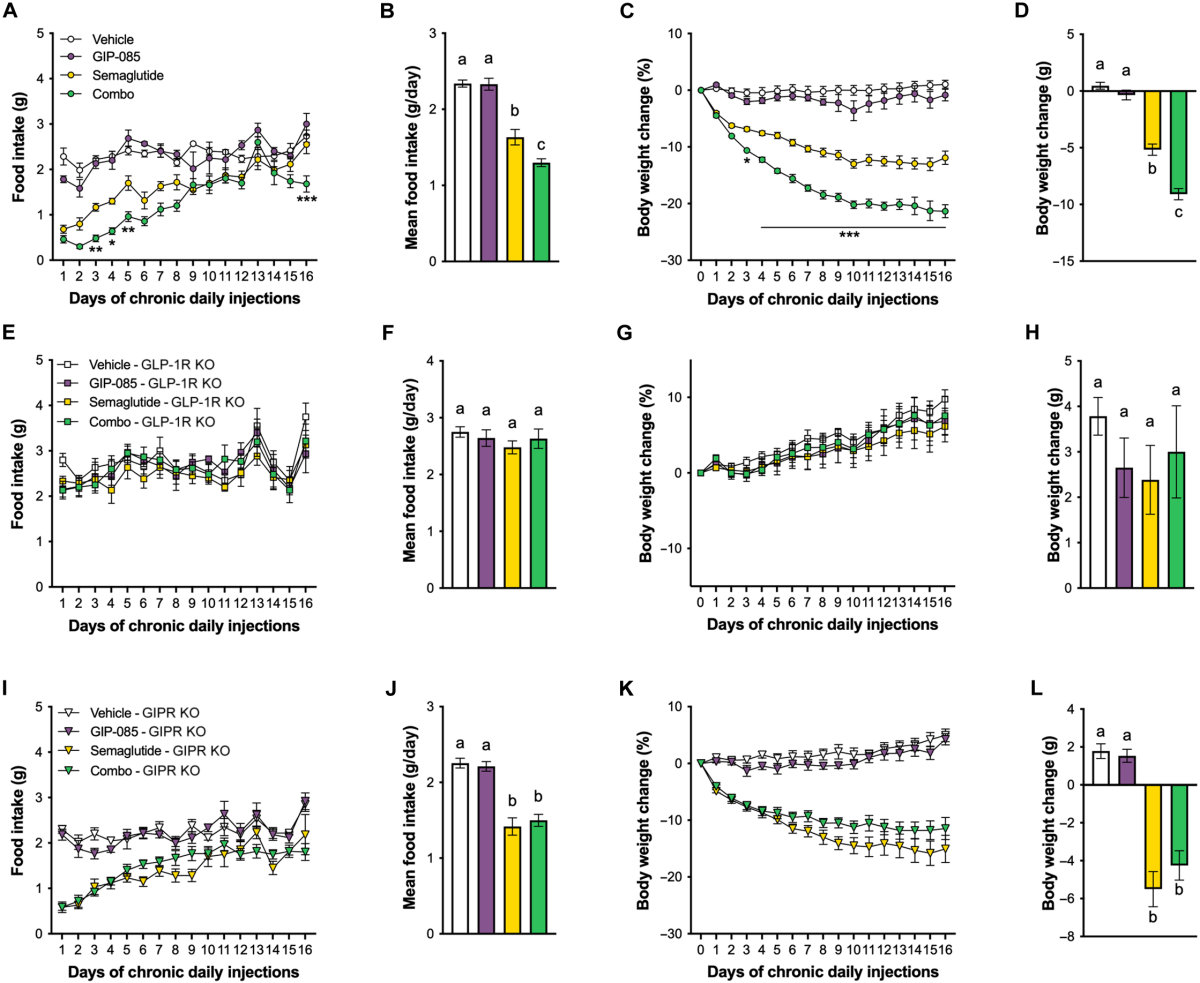
GIP agonism enhances semaglutide-induced anorexia and body weight loss in obese mice. (**A**) Chronic GIPA/GLP-1A dual treatment shows superior weight loss compared to semaglutide alone in DIO wild-type mice (GIP-085: 300 nmol/kg; semaglutide: 3 nmol/kg; *n* = 5 to 6 per group). (**B**) Mean daily food intake during the entire treatment period in DIO wild-type animals (*n* = 5 to 6 per group). (**C**) GIPA enhances GLP-1A–induced anorexia without having any effects on food intake alone in DIO wild-type mice (*n* = 5 to 6 per group). (**D**) Net body weight change at the end of the treatment period (*n* = 5 to 6 per group). (**E**) The anorectic effects of chronic semaglutide and dual semaglutide/GIP-085 treatments are absent in DIO GLP-1R KO mice (*n* = 6 per group). (**F**) Mean daily food intake during the entire treatment period in DIO GLP-1R KO mice (*n* = 6 per group). (**G**) No changes in body weight occurred following chronic treatments in DIO GLP-1R KO mice (*n* = 6 per group). (**H**) Net body weight change at the end of the treatment period (*n* = 6 per group). (**I**) GIP-085 treatment fails to enhance the anorectic effects of semaglutide in DIO GIPR KO mice (*n* = 6 per group). (**J**) Mean daily food intake during the entire treatment period in DIO GIPR KO animals (*n* = 6 per group). (**K**) No synergistic body weight lowering effects occurred in DIO GIPR KO mice following dual treatment (*n* = 6 per group). (**L**) Net body weight change at the end of the treatment period (*n* = 6 per group). All data expressed as means ± SEM. Data in (A), (C), (E), (G), (I), and (K) were analyzed with repeated measures two-way ANOVA followed by Tukey’s post hoc tests (*P < 0.05; **P < 0.01; ***P < 0.001). Only significant differences between semaglutide and combo treatment were annotated to enhance clarity. Data in (B), (D), (F), (H), (J), and (L) were analyzed with one-way ANOVA followed by Tukey’s post hoc tests. Means with different letters are significantly different from each other (*P* < 0.05).

We first tested whether chronic administration of the long-acting GIP analog GIP-085 alone or in combination with semaglutide promoted hypophagia and body weight loss in diet-induced obese (DIO) mice. We selected GIP-085 due to its long half-life, which makes it an ideal match for the long-acting GLP-1 analogs used in our studies. In line with previous reports using GIP analogs (*11*, *25*–*27*), daily systemic administration of GIP-085 had no major effects on food intake (Fig. 1, A and B) or body weight (Fig. 1, C and D, and fig. S1A). However, GIP-085 enhanced semaglutide-induced anorexia, leading to a greater body weight loss compared to semaglutide alone. The observed body weight effects are mainly due to loss of fat mass (fig. S1D). To confirm the specificity of GIP-085 and semaglutide action at the GIPR and GLP-1R, respectively, we conducted the same experiment in GIPR KO and GLP-1R KO DIO mice. No changes in food intake (Fig. 1, E and F), body weight (Fig. 1, G and H, and fig. S1B), or body composition (fig. S1E) occurred following chronic treatments in DIO GLP-1R KO mice. Similarly, no enhancement of hypophagia (Fig. 1, I and J) and body weight (Fig. 1, K and L, and fig. S1C) or fat mass (fig. S1F) loss occurred in DIO GIPR KO mice following dual treatment compared to semaglutide alone, indicating that GIP-085 failed to enhance the anorectic and body weight lowering properties of semaglutide in this model and indirectly validating the lack of cross-reactivity at the GLP-1R of GIP-085 in vivo.

### GIPR agonism attenuates GLP-1R–induced illness behaviors in obese rats

**Fig. 2.**
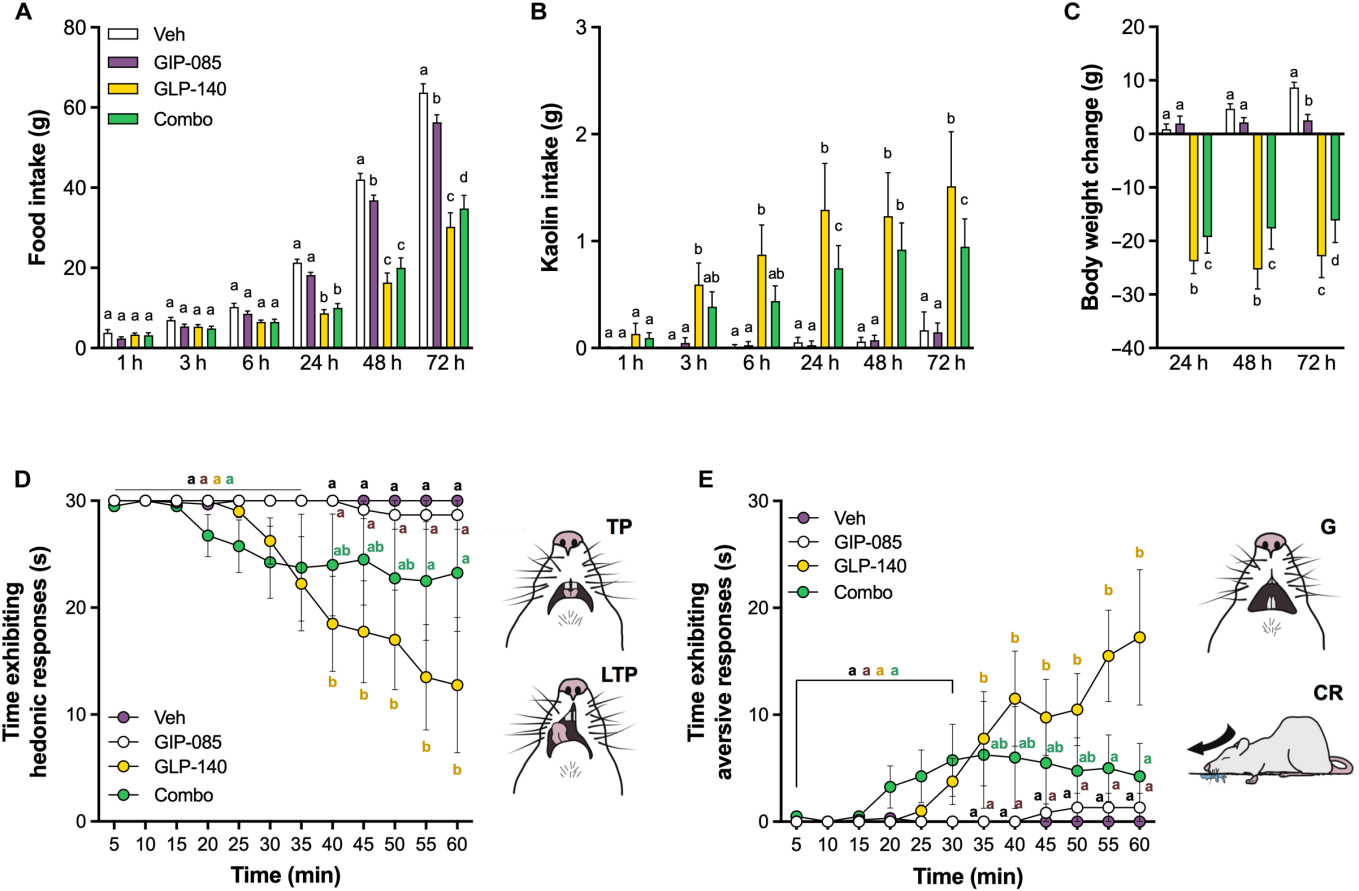
GIPR agonism attenuates GLP-1R–induced illness behaviors in obese rats. (**A**) Food intake of DIO rats following treatment with GIP-085 (300 nmol/kg), the long-acting GLP-1R agonist GLP-140 (1000 nmol/kg), combo, or vehicle (Veh). The anorectic effect of GLP-140 is partially reduced by GIP-085 cotreatment (ip, *n* = 15 per group). h, hours. (**B**) GLP-140 treatment causes significant kaolin intake. GIP-085 cotreatment attenuates kaolin intake induced by GLP-140 in DIO rats (*n* = 15 per group). (**C**) GIP-085 cotreatment reduces GLP-140–induced body weight loss (*n* = 15 per group) in DIO rats. (**D**) Whereas GIP-085:food association did not affect hedonic responses [i.e., tongue protrusions (TP) and lateral tongue protrusions (LTP)] compared to controls (300 nmol/kg, GIP-085; *n* = 6), GLP-140:food pairing significantly reduced the time spent expressing hedonic affective responses (1000 nmol/kg, GLP-140; *n* = 4). Cotreatment with GIP-085 (*n* = 4) was able to prevent the reduction in hedonic affective responses caused by GLP-140. (**E**) Conversely, GIP-085:food pairing did not condition any aversive reaction (300 nmol/kg, GIP-085; *n* = 6), whereas GLP-140:food association lead to significant increase in aversive responses (1000 nmol/kg, GLP-140; *n* = 4). GIP-085/GLP-140–treated rats (*n* = 4) did not show any significant increase in time spent exhibiting aversive responses [i.e., gapes (G) and chin rubs (CR)] compared to vehicle and GIP-085–treated animals (*n* = 6). All data expressed as means ± SEM. Data in (A) to (C) were analyzed with repeated measures two-way ANOVA followed by Tukey’s post hoc tests. Data in (D) and (E) were analyzed with two-way ANOVA followed by Tukey’s post hoc tests. Means with different letters are significantly different from each other (*P* < 0.05).

On the basis of our previous results indicating GIPR activation being antiemetic in lean rats against the preclinical GLP-1R agonist GLP-140 (*27*), we extended our investigation and tested the ability of GIP-085 to counteract the malaise induced by GLP-1R activation by measuring food intake, body weight, and kaolin consumption in obese rats following a single injection of the preclinical GLP-1R agonist GLP-140 (*27*). Because rodents lack the emetic reflex (*28*), pica behavior (i.e., ingestion of nonnutritive substances such as kaolin clay) is used in rats as a validated proxy for ongoing nausea/malaise (*29*–*31*) in response to treatments that induce nausea and vomiting in humans, including GLP-1 analogs. Because mice lack the ability to engage in pica behaviors reliably and quantifiably, the rat represents the superior model for this assessment. The dose of GLP-140 (i.e., 1000 nmol/kg) was selected based on published studies showing reliable induction of kaolin consumption and anorexia with lower inter-animal variability at this high dose relative to more moderate doses, which show greater variability in illness behaviors often observed in this species (*27*). Similar to what has been previously demonstrated in lean animals, GLP-140 treatment induced anorexia in obese animals (Fig. 2A), leading to an average 24-hour food intake reduction of 60 ± 3% relative to vehicle-treated controls. Rats treated with GLP-140 showed marked kaolin consumption as early as 3 hours postinjection, preceding the onset of the anorectic response and remaining significantly higher at all subsequent time points compared to controls (Fig. 2B). Anorexia induced by GLP-140 administration was associated with significant body weight loss 24 hours posttreatment (Fig. 2C). In line with previous reports (*27*, *32*), GIP-085 treatment alone had only minor effects on feeding and did not significantly induce kaolin intake or body weight loss. However, when coadministered with GLP-140, GIP-085 was able to reduce kaolin consumption driven by GLP-1R activation and it also significantly attenuated GLP-140–induced anorexia at 72 hours, resulting in a 7- ± 6-g significant attenuation of GLP-140–induced body weight loss. In addition, we conducted a follow-up study in which animals were treated with a low dose of semaglutide (10 nmol/kg) with or without GIP-085, yielding similar results (fig. S2).

We also hypothesized that GIP-085 cotreatment would attenuate the aversive conditioning induced by GLP-140. By measuring the change in the pattern of highly stereotyped, oral-facial responses from hedonic to aversive (*33*), this approach also determines the presence of a visceral malaise state, which can condition long-term feeding effects. Following GLP-140 or GIP-085 or GLP-140/GIP-085 treatment, rats received an orally delivered palatable taste every 5 for 60 min to reveal the rapid formation of an affective taste aversion in a single session (*34*). The analysis revealed that associating a palatable stimulus with GIP-085 did not condition a food aversion. The affective response to the food stimulus in GIP-085–treated rats was exclusively hedonic (Fig. 2D), entirely lacking disgust-aversive responses (Fig. 2E) and did not differ from vehicle treatment at any time point measured. In contrast, a single GLP-140–taste association was sufficient to rapidly change (i.e., within minutes) the affective response to food from hedonic to disgust/aversive via a conditioning process. This association significantly increased the time spent displaying disgust-aversive responses (Fig. 2E) and reduced time spent displaying hedonic responses (Fig. 2D) compared to GIP-085– and vehicle-treated rats. Cotreatment with GIP-085 effectively prevented the shift in affective responses from primarily hedonic to mostly aversive induced by GLP-140 (Fig. 2, D and E). Overall, these data expand and validate previous results using a complementary approach and further highlight the beneficial effects of GIPR agonism in attenuating malaise induced by GLP-1 ligands.

### Tirzepatide induces less kaolin consumption compared to semaglutide in rats

**Fig. 3.**
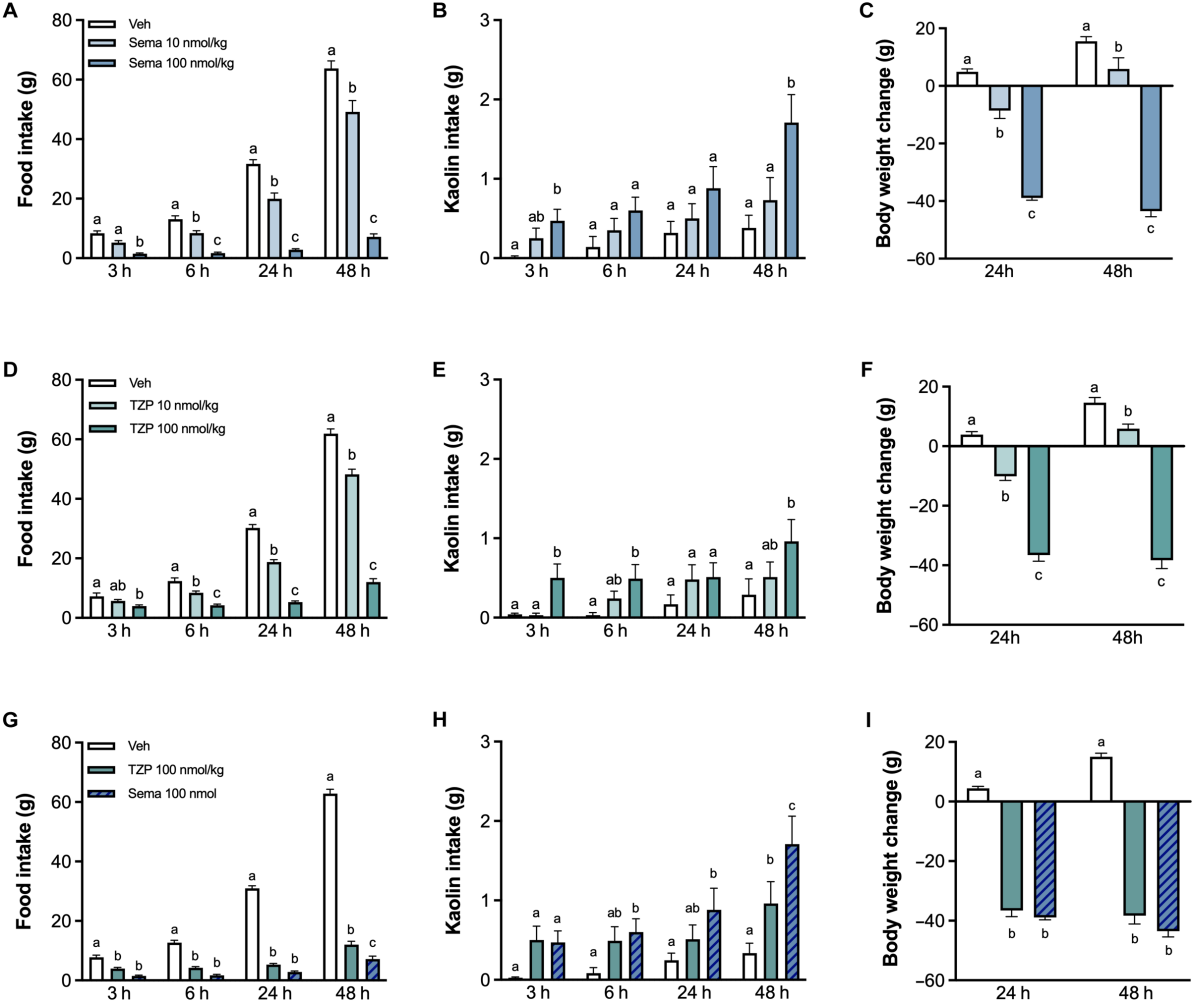
Tirzepatide induces less kaolin consumption in rats compared to equipotent doses of semaglutide. (**A**) Semaglutide administration dose-dependently suppresses food intake in rats (10 and 100 nmol/kg, ip; *n* = 10 per group). (**B**) The highest tested dose of semaglutide causes significant malaise (i.e., kaolin intake) in rats (100 nmol/kg, ip; *n* = 10 per group). (**C**) Semaglutide administration causes body weight loss in a dose-dependent manner (10 and 100 nmol/kg, ip; *n* = 10 per group). (**D**) Tirzepatide (TZP) dose-dependently suppress food intake (10 and 100 nmol/kg, ip; *n* = 10 per group). (**E**) Systemic administration of tirzepatide (100 nmol/kg) causes significant kaolin intake (ip, *n* = 10 per group). (**F**) Tirzepatide dose-dependently suppresses body weight (10 and 100 nmol/kg, ip; *n* = 10). (**G**) Tirzepatide and semaglutide induce comparable hypophagic responses in rats compared to controls (100 nmol/kg, ip; *n* = 10 to 20 per group). (**H**) Semaglutide administration induces higher kaolin consumption than tirzepatide treatment (100 nmol/kg, ip; *n* = 10 to 20 per group). (**I**) Tirzepatide and semaglutide cause similar body weight loss (100 nmol/kg, ip; *n* = 10 to 20 per group). All data expressed as means ± SEM. Data in (A) to (F) were analyzed with repeated measures two-way ANOVA followed by Tukey’s post hoc tests. Data in (G) to (I) were analyzed with repeated measures mixed-effects model followed by Tukey’s post hoc tests. Means with different letters are significantly different from each other (*P* < 0.05).

Clinical data suggest that tirzepatide treatment could be associated with a lower incidence of nausea and emesis in patients compared to semaglutide [see (*9*, *10*, *22*) for review]. To test this hypothesis, we first conducted a study in which rats were treated with equimolar doses of semaglutide and tirzepatide. All selected doses induced hypophagia (Fig. 3, A and D) and body weight loss (Fig. 3, C and F) in a similar dose-dependent manner. In addition, both drugs, at their highest doses significantly caused kaolin intake in rats (Fig. 3, B and E). When directly compared and analyzed, both high doses induced comparable hypoghagic responses (Fig. 3G) that lead to similar body weight loss (Fig. 3I). However, the magnitude of the increase in kaolin consumption caused by semaglutide (100 nmol/kg) was significantly different than controls and significantly higher than that caused by tirzepatide (100 nmol/kg) (Fig. 3H), thus suggesting a more favorable tolerability of tirzepatide compared to semaglutide.

### Semaglutide improves glucoregulation, induces anorexia and body weight loss, and induces emesis in shrews, an effect that is blocked by GIPR agonism

**Fig. 4.**
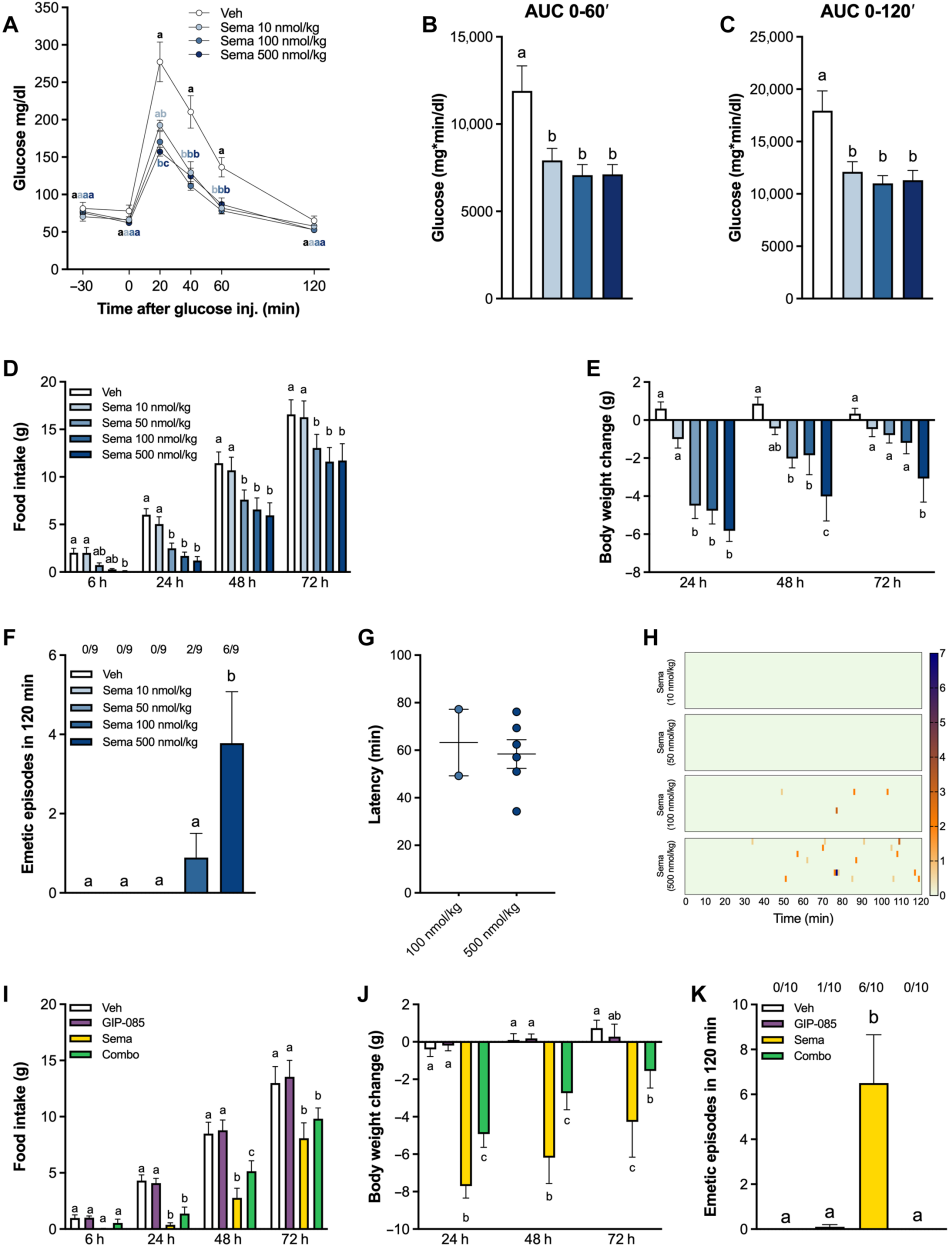
Semaglutide dose-dependently improves glucoregulation, induces anorexia and body weight loss, and induces emesis in shrews, which is blocked by GIPR agonism. (**A**) Semaglutide (10, 100, and 500 nmol/kg) dose-dependently suppresses blood glucose levels following glucose administration in shrews (2 g/kg, ip; *n* = 8 per group). (**B**) Glucose AUC from 0 to 60 min after treatment (*n* = 8 per group). (**C**) Glucose AUC from 0 to 120 min after treatment (*n* = 8 per group). (**D**) Systemic semaglutide administration (10, 50, 100, and 500 nmol/kg, ip) causes anorexia in a dose-dependent manner (*n* = 8 per group). (**E**) Semaglutide dose-dependently induces body weight loss (10, 50, 100, and 500 nmol/kg, ip; *n* = 8 per group). (**F**) Semaglutide administration causes emesis in a dose-related manner in shrews (10, 50, 100, and 500 nmol/kg, ip; *n* = 9 per group). The proportion of animals exhibiting emesis is indicated above each treatment group. (**G**) Latency to the first emetic episodes of shrews that exhibited emesis. (**H**) Heatmaps showing the latency, number, intensity, and frequency of emesis following different doses of semaglutide for each individual animal. (**I**) Semaglutide-induced anorexia is partially attenuated by GIP-085 cotreatment (semaglutide: 500 nmol/kg, GIP-85: 300 nmol/kg, ip; *n* = 8 per group). (**J**) GIP-085 cotreatment attenuates semaglutide-induced weight loss (semaglutide: 500 nmol/kg, GIP-85: 300 nmol/kg, ip; *n* = 8 per group). (**K**) The profound emesis induced by semaglutide (500 nmol/kg, ip) is completely blocked by GIP-085 (300 nmol/kg, ip) cotreatment (*n* = 10 per group). The proportion of animals exhibiting emesis, is indicated above each treatment group. Data expressed as means ± SEM. Data in (A), (D), (E), (I), and (J) were analyzed with repeated measures two-way ANOVA, followed by Tukey’s post hoc tests. Data in (B) and (C) were analyzed with one-way ANOVA, followed by the Tukey’s post hoc test. Data in (F, K) were analyzed with repeated measures one-way ANOVA, followed by Tukey’s post hoc tests. Means with different letters are significantly different from each other (*P* < 0.05).

To further investigate the capacity of GIPR agonism in alleviating semaglutide-induced emesis and to compare the differences in emetogenic potential between semaglutide and tirzepatide, the house musk shrew model was used. The house musk shrew (*Suncus murinus*) is an established preclinical model for emesis due to its capability to vomit and an emetic profile similar to humans (*35*–*40*). This model organism exhibits hypoglycemia, anorexia, and emetic sensitivity induced by several existing GLP-1R agonists (*38*, *41*–*43*), providing a validated emetic species model of GLP-1R signaling. Because the effects of semaglutide had not been previously assessed in this model, we first evaluated the effects of various doses of semaglutide on glucose levels following an intraperitoneal glucose tolerance test (IPGTT). Results demonstrated that all selected doses of semaglutide significantly reduced glucose levels in a dose-dependent manner at 20 min post-glucose injection (Fig. 4A), resulting in similar total overall blood glucose suppressions compared to control animals, as reflected by the area under the curve (AUC) 0 to 120′ and AUC 0 to 60′, respectively (Fig. 4, B and C).

We then tested the ability of semaglutide to induce anorexia and body weight loss. Although less robust than what was observed in rats, the observed effects aligned with previous reports investigating hypophagic effects of other GLP-1R agonists in this model. Systemic administration of semaglutide produced anorexia in a dose-dependent manner in the shrew (Fig. 4D), leading to significant body weight loss up to 72 hours after the highest dose tested (Fig. 4E). Last, we tested the ability of semaglutide to induce emesis in shrews across the same dose range. Results clearly show that semaglutide dose-dependently induced emesis with most of the shrews experiencing emesis after semaglutide (500 nmol/kg) dosing (Fig. 4F). Twenty-two percent of the animals exhibited emesis upon administration with the third (100 nmol/kg) and 67% with the highest dose tested (500 nmol/kg). All doses of semaglutide that induced emesis did so with an average latency of 63 ± 14 and (100 nmol/kg) and 58 ± 6 min (500 nmol/kg), respectively (Fig. 4G). The emetic profiles of each animal following administration of different doses of semaglutide are represented in Fig. 4H.

We subsequently analyzed the effects of combinatorial GIP-085/semaglutide agonism on feeding, body weight, and emesis in shrews. Although semaglutide treatment alone induced anorexia (Fig. 4I) and body weight loss (Fig. 4J), GIP-085 cotreatment partially reduced hypophagia, and consequently, this resulted in a marginal yet significant attenuation of the body weight lowering effect of semaglutide treatment at 24 and 48 hours. GIP-085 cotreatment was able to completely prevent semaglutide-induced emesis (Fig. 4K). Although 60% of the shrews experienced emesis following semaglutide administration, no emesis was observed after GIP-085/semaglutide cotreatment. Overall, these results clearly demonstrate in an emetic species model the ability of GIP-085 to block the emetogenic properties of semaglutide.

### Tirzepatide dose-dependently enhances glucoregulation and induces anorexia and body weight loss, without causing emesis in shrews

**Fig. 5.**
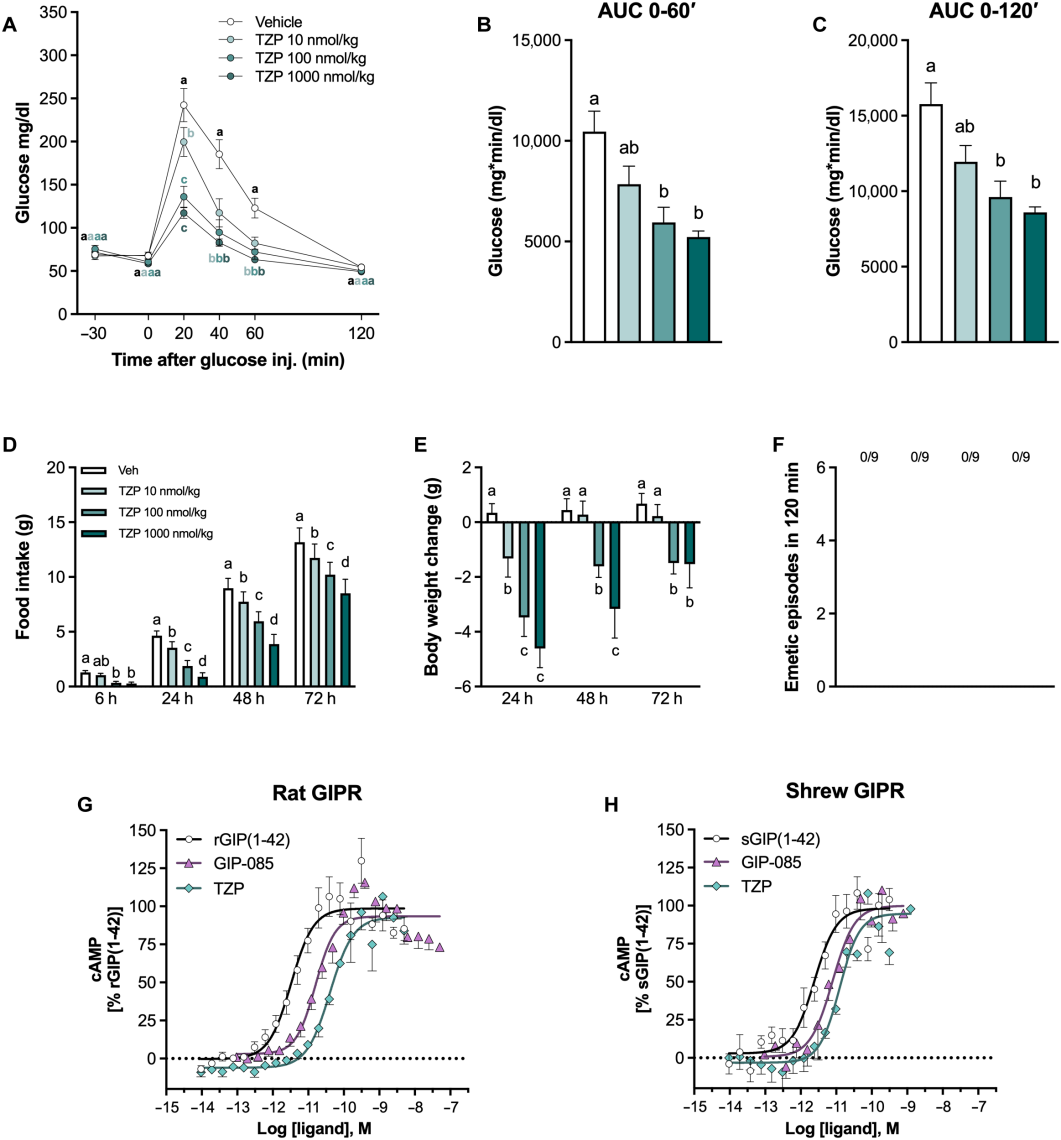
Tirzepatide dose-dependently enhances glucoregulation, induces anorexia, and body weight loss, without causing emesis in shrews. (**A**) Tirzepatide (10, 100, and 1000 nmol/kg) dose-dependently suppresses blood glucose levels following glucose administration in shrews (2 g/kg, ip; *n* = 7 per group). (**B**) Glucose AUC from 0 to 60 min after treatment (*n* = 7 per group). (**C**) Glucose AUC from 0 to 120 min after treatment (*n* = 7 per group). (**D**) Tirzepatide administration (10, 100, and 1000 nmol/kg, ip) induces hypophagia in a dose-dependent manner (*n* = 9 per group). (**E**) Tirzepatide dose-dependently induces body weight loss (10, 100, and 1000 nmol/kg, ip; *n* = 9). (**F**) In contrast to semaglutide administration, no emesis occurred in shrews after receiving tirzepatide (10, 100, and 1000 nmol/kg, ip; *n* = 9 per group). The number of animals exhibiting emesis, expressed as a fraction of the total number of animals tested, is indicated above each treatment group. (**G**) Concentration-response curves (CRCs) for cAMP accumulation in response to either rat GIP (1-42), GIP-085, or tirzepatide using HEK cells transiently expressing rat GIPR (*n* = 3 independent experiments performed with eight replicates at each point). (**H**) CRCs for cAMP accumulation in response to either shrew GIP (1-42), GIP-085, or tirzepatide using HEK cells transiently expressing shrew (*S. etruscus*) GIPR (*n* = 3 independent experiments). Data expressed as means ± SEM. Data in (A), (D), and (E) were analyzed with repeated measures two-way ANOVA, followed by Tukey’s post hoc tests. Data in (B) and (C) were analyzed with one-way ANOVA, followed by the Tukey’s post hoc test. Data in (F) were analyzed with repeated measures one-way ANOVA, followed by Tukey’s post hoc tests. Means with different letters are significantly different from each other (*P* < 0.05).

To expand our current dataset in rats and provide direct indications on the glucoregulatory, anorexigenic and emetogenic potential of tirzepatide in shrews, similar dose response studies were conducted. Results showed that tirzepatide dose-dependently suppressed glucose levels following and IPGTT (Fig. 5, A to C), indicating the ability of tirzepatide to enhance glucoregulation in this model as well. Furthermore, systemic administration of tirzepatide produced significant hypophagia and body weight loss in shrews in a dose-dependent manner and similar magnitude to what we observed following semaglutide treatment (Fig. 5, D and E). Although semaglutide induced profound emesis, animals receiving all tested doses of tirzepatide showed a complete absence of emesis. Together, these results indicate that tirzepatide and semaglutide share similar glucoregulatory, hypophagic, and body weight reducing effects in the shrew, but they substantially diverge in terms of tolerability and in their ability to cause emesis and nausea.

Last, to confirm that tirzepatide and GIP-085 engage the rat and shrew GIPR with similar potency, we performed cyclic adenosine monophosphate (cAMP) binding assays in human embryonic kidney (HEK) 293 cells transiently transfected to express either the rat or shrew GIPR. Because of the absence of a fully sequenced musk shrew genome, we used the Etruscan shrew (*Suncus etruscus*) GIPR gene as it belongs to the same family and genus and is considered one of the closest related shrew species to the musk shrew (*S. murinus*) (*44*). Our results show that, in HEK293 cells expressing rGIPR, GIP-085 and tirzepatide exhibited comparable potency with a median effective concentration (EC_50_) of 0.016 and 0.04 nM, respectively. The native rat GIP has an EC_50_ of 0.0035 nM (Fig. 5G). A similar kinetic profile for cAMP production and decay was observed at sGIPR following GIP-085 (EC_50_ = 0.007 nM) and tirzepatide administration (EC_50_ = 0.013 nM), which was comparable to the native shrew GIP peptide (EC_50_ = 0.002 nM, Fig. 5H). Overall, these in vitro studies suggest that both GIP-085 and tirzepatide mimic the actions of native GIPs at both rat and shrew GIPRs with similar potency. Although the effects of tirzepatide and GIP-085 on the human GIPR were not assessed in the current study, comparable EC_50_ values were observed in human adipocytes expressing the hGIPR (*45*), supporting the translational relevance of these findings.

## DISCUSSION

Among the various current therapeutic options, the long-acting GLP-1R agonist semaglutide and the GLP-1R/GIPR agonist tirzepatide are considered the most efficacious treatments for obesity and T2DM today due to their ability to both normalize glycemia and reduce food intake and body weight [see (*1*–*3*) for review]. In the current study, we investigated the effects of multiple doses and combinations of GLP-1R, GIPR, and GLP-1R/GIPR agonists on energy homeostasis and several malaise behaviors in multiple species. We showed here that GIPR agonism blocks emesis in shrews and attenuates other malaise behaviors in rats elicited by GLP-1R activation while maintaining reduced food intake and body weight loss and improved glucose tolerance. These findings underscore the therapeutic potential of combined pharmaceutical strategies targeting both incretin systems and highlight contrasting results when looking at secondary endpoints compared to GLP-1R agonism alone.

Current data from mice clearly demonstrate the importance of GIPR signaling in enhancing semaglutide-induced hypophagia and body weight/fat mass reductions, aligning with previously published clinical data (*12*, *15*–*21*). The results from the mouse KO models also provide behavioral validation in vivo, confirming the lack of cross-reactivity at the GLP-1R for GIP-085 and at the GIPR for semaglutide, respectively. Although not directly tested, these data also seem to reject the hypothesis of compensatory effects of each of these two systems on food intake and body weight. Although an incretin compensation has been observed in terms of enhanced insulin secretion following genetic elimination of the receptor for the other (*46*), this phenomenon does not seem to occur with respect to food intake and body weight in mice. It is also plausible that differences in dosage and in the pharmacodynamic and pharmacokinetic profiles of the GIPR and GLP-1R agonists used could explain the lack of a compensatory effect in the current study.

A growing body of clinical literature suggests differences between the incidence of nausea and emesis following semaglutide and tirzepatide treatments, with the latter being better tolerated by patients [see (*9*, *10*, *22*) for review]. However, the lack of standardized protocols for evaluating/reporting these adverse events, combined with the challenge of accurately assessing subjective feelings of nausea in patients and variations in dose-escalation protocols and participant selection criteria across different studies, makes drawing definitive conclusions difficult. In addition, one could argue that some of these effects might be due to the unique intrinsic properties of tirzepatide, which is a mixed GIP/GLP-1 analog with biased agonism at the GLP-1R (*23*, *24*) and may have different profiles of pharmacodynamics, pharmacokinetics, receptor pharmacology, and biodistribution in central nervous system (CNS) nuclei compared to semaglutide. Therefore, (i) the use of multiple species and paradigms to assess behaviors indicative of malaise and (ii) the separate administration of GIP and GLP-1 analogs in addition to tirzepatide were vital to resolving the clinical dichotomy discussed above.

The ability of GIP-085 to completely prevent emesis caused by semaglutide in shrews was particularly notable, but perhaps even more so were the data obtained following tirzepatide, denoting a complete absence of any emetic episode in shrews at the doses tested. It has been demonstrated that tirzepatide exhibits a bias at the GLP-1 receptor to favor cAMP generation over β-arrestin recruitment, coincident with a weaker ability to drive GLP-1R internalization compared to native GLP-1 (*23*, *24*). In addition, although some evidence suggests that both compounds do not robustly penetrate the blood-brain barrier (BBB) (*47*–*49*), they might differ in their ability to directly reach and affect specific brain regions that either lack a functional BBB or are located near to the brain ventricles such as the area postrema and the adjacent the nucleus of the solitary tract (collectively referred as AP/NTS from now on). Although we cannot exclude that these intrinsic properties of tirzepatide might directly or indirectly contribute its tolerability profile, our combined data strongly suggest that the better tolerability profile of tirzepatide is not due to its intrinsic properties, but it is rather caused, at least in part, by the actions of tirzepatide at the GIPR.

The beneficial effects of GIPR agonism combined with GLP-1R agonism, whether coadministered as GIP-085 or delivered in the form of tirzepatide, were less profound in rats. Although GIPR agonism prevented the formation of conditioned aversive reactions induced by GLP-1R activation, kaolin consumption following GIP-085/semaglutide and tirzepatide treatment were still significantly elevated compared to vehicle-treated animals. Because the in vitro kinetics of cAMP modulation showed nearly identical profiles for both ligands at sGIPR and rGIPR, it is unlikely that species-specific signaling dynamics play a major role. Given that kaolin intake (i.e., pica behavior) can be viewed as a proxy of malaise and nausea in rats (*50*), these results could instead suggest that GIPR agonism may be more efficacious as an antiemetic than as an anti-nausea therapeutic. The interpretation of our current dataset is in line with the clinical data reporting a more substantial reduction in vomiting versus nausea in patients receiving tirzepatide compared to patients receiving semaglutide (emesis, sema 1 mg: 8.3 to 13.4% versus tirzepatide 5 mg: 3.3 to 5.7%, sema 2.4 mg: 21.8% versus tirzepatide 10 mg: 2.5 to 8.5%; nausea, sema 1 mg: 17.9 to 32.1% versus tirzepatide 5 mg: 11.6 to 17.4%; sema 2.4 mg: 33.7% versus tirzepatide 10 mg: 13.2 to 19.2%) (*22*). Similar profiles could also be observed in other medical fields following administration of classic antiemetic. A good example is perhaps the oncology field, in which thanks to the use of various antiemetics (e.g., ondansetron), emesis is relatively well managed, whereas nausea remains poorly controlled (*51*, *52*).

The development of treatment strategies that target both the GIPR and the GLP-1R system represents a significant breakthrough in the fight against obesity and T2DM. However, although the roles of the central GLP-1R system have been extensively studied in the past decades [see (*1*, *53*–*55*) for review], the roles of the GIPR system in the modulation of energy homeostasis, the central mechanisms of action of GIPR analogs, and the mechanisms behind the augmented metabolic effects upon coadministration with GLP-1R agonists compared to mono–GLP-1R agonism remain elusive. Although GIPR expression within the CNS has been documented in the early 1990s (*56*), the central actions of GIP ligands on feeding behaviors have been only recently started to be investigated. Recent evidence demonstrated that GIPR agonists depend on GIPR signaling in inhibitory AP/NTS GABAergic neurons to decrease body weight and food intake (*27*, *57*, *58*). The antiemetic effects of GIPR agonists also seem to depend on these neurons, as demonstrated by the ability of selective hindbrain GIPR activation to antagonize GLP-1R–induced kaolin intake in rats, thus replicating the effects of systemically delivered GIP agonists (*27*). Moreover, GIP administration and selective activation of AP/NTS GABAergic neurons both suppress conditioned taste avoidance caused by emetic stimuli (*59*). In line with these behavioral effects, GIPR agonism significantly reduced neuronal activation in the AP/NTS caused by both GLP-1 analogs and the chemotherapy agent cisplatin in shrews and rats, respectively, further supporting a role of the hindbrain in the antiemetic action of GIPR agonism (*27*, *32*). In addition, data generated by various labs using the single-nucleus sequencing platform provided further evidence by identifying transcriptomically distinct populations of GLP-1R^+^ and GIPR^+^ neurons in the AP/NTS (*27*, *32*, *60*, *61*). These results showed that only a limited number of neurons coexpress both GIPR and GLP-1R, highlighting that GIPR agonism is engaging a distinct first-order neuronal circuit from those activated by GLP-1R agonism within the AP/NTS. Whether GIPR-expressing neurons located in other areas of the brain, such as the hypothalamus, also contribute to the beneficial metabolic effects of GIPR agonists remains an area of future investigations.

It remains to be evaluated whether GIPR within the brain play a physiological role in the control of food intake and the specific source(s) of the ligand. It is also unclear whether the endogenous GIP is involved in the regulation of emesis and nausea. However, given the short half-life of both endogenous ligands, it is unlikely that GIP and its receptor system evolved to prevent nausea and emesis caused by its sister incretin GLP-1. Nonetheless, treatments in the form of bispecific molecules engineered by conjugating a GIPR antagonizing antibody with a GLP-1R agonist (i.e., AMG 133) lead to significant nausea and emesis, with 100% of patients reporting nausea upon treatment with multiple ascending doses (*62*). On the basis of these outcomes one can speculate that inhibition of endogenous GIPR signaling exacerbates nausea and emesis caused by the pharmacological activation of GLP-1R in the brain; however, more studies are necessary to assess this theory.

### Limitation of the study

In conclusion, our data gathered in three preclinical species do not only demonstrate that GIPR chronic activation enhances the food intake and body weight suppression of semaglutide in obese mice, but it also blocks emesis and attenuates illness-like behaviors (i.e., pica and conditioned aversive responses) elicited by GLP-1R activation in shrews and rats, respectively. A limitation of the conclusions drawn from the current set of data rests on the fact that analyses were acute/subchronic in nature and additional studies assessing the long-term tolerability profiles of the compounds tested are needed. Emerging evidence indicates that also GIPR antagonism could represent a viable strategy to suppress adiposity and body weight (*11*, *62*, *63*) [see (*64*) for review]. These seemingly paradoxical and contradictory effects are admittedly hard to reconcile and are a current source of debate. It is possible that different GIPRs expressed through the body, including the brain, might be responsible for the mediation of different and potentially antagonizing aspects of the GIP effects. Although this is an interesting area of study, it falls beyond the scope of the current manuscript. Although the effects of GIPR antagonism on emesis has not been specifically investigated, it is, however, very unlikely that the antiemetic effects of GIPR agonism are due to functional antagonism, given the acute nature of the most of our studies and the rapid onset of GIPR agonism effects.

In most of the current rat and shrew combination experiments, we intentionally administered high doses of GLP-1 analogs to reliably induce malaise, thereby overcoming the variability in illness behaviors often observed at moderate doses. This approach allowed for a more effective assessment of whether GIP agonism could attenuate nausea/emesis; however, it also limited the ability to evaluate potential synergistic effects on food intake and body weight, which were observed in the subchronic mouse experiment and have been reported by other groups using lower chronic doses. It is worth mentioning, however, that these synergistic effects of GIPR agonism tend to be modest initially and only become evident and statistically significant under chronic conditions after multiple days of treatment (*12*, *25*, *65*). One could also speculate that the unexpected small but significantly higher food intake and reduced body weight loss observed following acute GIP/GLP-1 administration compared to GLP-1 monotreatment may reflect an overall reduction in malaise experienced by the animals during the acute phase of treatment, when the incidence and severity of nausea and emesis are typically highest. Further studies are needed to clarify this observation.

Another limitation is represented by the absence of a sequenced musk shrew genome, which substantially restricts the genetic and molecular tools available to assess the role of specific neuronal populations in this model. Despite these limitations, current results further strengthen the value of combinatorial GLP-1/GIP treatments as these approaches could not only increase efficacy in treating obesity and diabetes, but they could also increase patient retention and potentially improve the therapeutic index of GLP-1R agonists.

## MATERIALS AND METHODS

### Experimental model details

All procedures were approved by the Institutional Care and Use Committee of the University of Pennsylvania and Eli Lilly and Company.

DIO adult male C57BL/6 mice (Taconic) C57BL/6 (*n* = 23, weighing ~43 g at arrival), GLP-1R KO (germline knockout line: 11549, *n* = 24, weighing ~38 g at arrival), and GIPR KO mice (germline knockout line: 2886, *n* = 24, weighing ~35 g at arrival) were used. Upon arrival, mice were individually housed and continued to be fed ad libitum on high-fat/high-sugar diet (60% fat diet, 12492, Research Diets).

Adult male Sprague-Dawley rats (Charles River) weighing ~250 to 270 g (*n* = 45) at arrival were housed under a 12-hour/12-hour light/dark cycle in a temperature-controlled and humidity-controlled vivarium. Rats were individually housed in hanging wire-bottom cage with ad libitum access to chow diet (Purina Lab Diet 5001) or, when noted, to high-fat/high-sugar diet (60% fat diet, 12492, Research Diets) and tap water and had ad libitum access to kaolin pellets (Research Diets, K50001). Rats were exposed to kaolin for at least 5 days prior to measuring kaolin consumption in pica testing. Kaolin intake and food consumption were measured simultaneously as the presence of kaolin in the cage, and its subsequent ingestion, has usually negligible effects on the hypophagic and body weight–lowering effects of the treatments.

Adult male shrews (*S. murinus*) weighing ~50 to 80 g (*n* = 84 total) were bred and maintained in the De Jonghe Lab (University of Pennsylvania). These animals were offspring from a colony previously maintained at the University of Pittsburgh Cancer Institute (C. Horn); a Taiwanese strain was derived from stock originally supplied by the Chinese University of Hong Kong. Shrews were singly housed in plastic cages (37.3 cm by 23.4 cm by 14 cm, Innovive) under a 12-hour/12-hour light/dark cycle in a temperature-controlled and humidity-controlled environment. Animal were fed ad libitum with a mixture of feline (75%, Laboratory Feline Diet 5003, Lab Diet) and mink food (25%, High Density Ferret Diet 5LI4, Lab Diet) and had ad libitum access to tap water except where noted.

Animals were habituated to single housing in their home cage and intraperitoneal injections at least 1 week prior to experimentation. All animals were naïve to experimental drugs and test prior to the beginning of the experiment. For most in vivo experiments, injections were administered using a within-subject design, Latin square design. The exceptions were chronic administration studies in mice and the glucose tolerance tests in rats, which were conducted using a between-subject design. For these experiments, animals were randomized into groups based on body weight to ensure balanced distribution across treatment conditions. In all experiments using a within-subject design, all experimental injections were separated by at least 7 days. In all feeding studies, injections were performed shortly before dark onset. All experiments were performed in accordance with the ARRIVE (Animal Research: Reporting of In Vivo Experiments) guidelines.

### Drugs and route of administration

GIPFA-085 (GIP-085), tirzepatide, and GLP-140 were designed and produced by Eli Lilly and Company. GIP-085, GLP-140, and semaglutide (Cayman Chemical) were dissolved in 40 mM tris-HCl buffer (pH 8) and 0.02% Tween 80. For the study in mice, all drugs were injected subcutaneously at a volume of 5 ml/kg of body weight. In rats, all drugs were injected intraperitoneally, at a volume of 1 ml/kg of body weight, except when noted. In shrews, all drugs were injected intraperitoneally at a volume of 10 ml/kg of body weight.

### Effects of chronic GIP-085, semaglutide, and GIP-085/semaglutide dual treatment on food intake body weight and body composition in DIO wild-type, GLP-1RKO, and GIPR KO mice

C57BL/6 wild-type (*n* = 23), GLP-1R KO (*n* = 24), and GIPR KO mice (*n* = 24) received daily subcutaneous administration of GIP-085 (300 nmol/kg), semaglutide (3 nmol/kg), GIP-085/semaglutide combo, or vehicle for 16 days. Food intake and body weight were manually measured daily. The average body weight at the start of the experiment was 43.7 ± 0.4 g for the wild-type group, 39.0 ± 0.6 g for the GLP-1R KO group, and 36.5 ± 0.4 g for the GIPR KO group. Body composition of mice was determined using quantitative nuclear magnetic resonance analysis (ECHO MRI, 3-1 Composition Analyzer; Echo Medical Systems). At the end of the experiment, the animals were euthanized by CO_2_.

### Effects of GIP-085, GLP-140, and GIP-085/GLP-140 dual treatment on food and kaolin consumption and body weight in obese rats

Kaolin intake [i.e., pica, a well-established as a model of nausea in rats (*50*)] was measured in addition to food intake. Rats kept on high-fat diet (*n* = 15, 720 ± 17 g), food consumption, kaolin intake, and body weight were measured after an intraperitoneal administration of GIP-085 (300 nmol/kg), GLP-140 (1000 nmol/kg), combo, or vehicle. Food and kaolin intake were measured at 1, 3, 6, 24, 48, and 72 hours postinjection, and body weight was measured at 0, 24, 48, and 72 hours. A within-subject paradigm was used, with each round of injections separated by 7 days.

### Effects of GIP-085, semaglutide, and GIP-085/semaglutide dual treatment on food and kaolin consumption and body weight in rats

In a cohort of rats (*n* = 15 per group, body weight ~ 460 ± 9 g), food consumption, kaolin intake, and body weight were measured after an intraperitoneal administration or after an intraperitoneal administration of GIP-085 (300 nmol/kg), semaglutide (10 nmol/kg), combo, or vehicle. Food and kaolin intake were measured at 1, 3, 6, and 24 hours postinjection and body weight was measured at 0 and 24 hours. A within-subject paradigm was used, with each round of injections separated by 7 days.

### Taste reactivity assessment of conditioned disgust/aversive affective reaction to food following GIP-085 and GLP-140 treatments

#### Intraoral cannula surgery

Obese rats (*n* = 10) were anesthetized [ketamine (90 mg/kg; Midwest Veterinary Supply), xylazine (2.7 mg/kg; Anased), and acepromazine (0.64 mg/kg; Midwest Veterinary Supply] and were implanted with a unilateral intraoral (IO) cannula consisting of polyethylene (PE-100) tubing heat flared to hold a Teflon washer on the proximal end and press fit with 19-gauge stainless steel tubing on the distal end according to protocol (*33*). Briefly, the intraoral cannula was implanted lateral to the first maxillary molar and then anchored to the skull with a head cap made from four set screws and dental acrylic. Postoperative antibiotic [Baytril (enrofloxin; 10 mg/kg, subcutaneously (sc)] and analgesic (meloxicam; 2 mg/kg, sc) were administered immediately after surgery and once daily for 3 days. Rats were given a limited amount of chow mash (∼50% powdered chow: 50% H_2_O) after surgery and for at least two following days before ad libitum access to hard chow.

#### Food stimuli, intraoral infusion habituation, intraoral food stimuli infusions, and video recording test sessions

Two different food stimuli served as conditioned stimuli in experiments using the taste reactivity test, saccharin [Acros Organics, 0.15% (w/v) in distilled water] or chicken stock (Fresh Market, diluted 1:1 with distilled water). These two distinct tastants, which qualities have nonoverlapping sensory qualities, were used to maximize the number of stimuli each animal was exposed to. Animals were first exposed to tastant #1 (either chicken stock or saccharin), followed by exposure to the other tastant (#2) 1 week later. The sequence of tastant presentation, as well as the tastant-drug pairing, were randomized and counterbalanced to avoid potential bias. Rats were naive to the stimulus selected on their initial test day exposure and weighed ~490 ± 13 g. Food stimulus–subcutaneous drug pairings were made as described below. Starting 1 day postoperatively until the start of the experiment, intraoral cannulas were flushed with water daily. Four days prior to the experiment, rats were habituated to the test chamber for 45 min twice a day. The day before the start of the experiment, rats underwent a mock session. They were placed in the chamber, given 5 min to acclimate and then infused with tap water for 30 s at the same rate (1 ml/min) as they would subsequently receive on test day. Taste reactivity habituation and testing were conducted in a cylindrical chamber with clear Plexiglas walls and floor. A mirror was mounted at a 45° angle just below the clear chamber floor, and a digital video camera (Toshiba Camileo H30 HD) was positioned facing the mirror on a tripod ∼35 cm away. The PE-100 infusion line connected the rat’s intraoral cannula to a 10-ml syringe secured to an infusion pump (Harvard Apparatus) that contained the food stimulus. On testing days, rats were injected with GIP-085 (300 nmol/kg) or vehicle and 30 min later received a second injection of GLP-140 (1000 nmol/kg) or vehicle. Rats were immediately connected to the infusion pump and placed in the test chamber. The experimenter activated the camera and tracked the animal ventral surface focusing on the oral-facial region. The first 30-s infusion was given at 5 min. Infusions then occurred every 5 min for 60 min.

#### Taste reactivity affective behavior analysis

Video files were viewed by a trained observer who was unaware of the stimulus or group assignment of the rat in each recording. Each 30-s infusion period of food-elicited responses was analyzed for every occurrence of oral-facial and other behavioral responses described under the rubric of taste reactivity (*33*, *66*), categorized into two affective reaction patterns to food: hedonic reactions and disgust/aversive reactions. The total time spent displaying behaviors comprising each affective response category was later quantified. The response components comprising hedonic and disgust/aversive categories have been described in detail elsewhere (*33*, *34*, *66*). Hedonic responses included tongue protrusions, mouth movements, and lateral tongue protrusions. Reactions comprising the disgust/aversive category included gapes, chin rubs, headshakes, forelimb flails, and paw treading.

### Effects of semaglutide and tirzepatide on food and kaolin consumption and body weight in rats

In two separate cohorts of rats (*n* = 10 per group, body weight ~ 460 ± 9 g), food consumption, kaolin intake, and body weight were measured after an intraperitoneal administration of vehicle, semaglutide (10 and 100 nmol/kg), or tirzepatide (10 and 100 nmol/kg), respectively. Food and kaolin intake were measured at 3, 6, 24, and 48 hours postinjection, and body weight was measured at 0, 24, and 48 hours. For each drug, a within-subject paradigm was used, with each round of injections separated by 7 days.

### Dose-response effects of semaglutide on glycemic control in shrews

The protocol for performing an IPGTT in shrews (*n* = 16) was previously described (*27*). Briefly, each shrews received intraperitoneal injection of semaglutide (10, 100, and 500 nmol/kg) or vehicle (10 ml/kg body weight) followed 30 min later by a glucose bolus injection [2 g/kg, intraperitoneally (ip)]. Blood glucose readings were taken at −30, 0, 20, 40, 60, and 120 min post-glucose administration. This study was conducted using a partial within-subject design, with each animal receiving two treatments separated by a 7-day interval.

### Dose-response effects of semaglutide on energy balance and emesis in shrews

We evaluated the effects on food intake and body weight of different doses of semaglutide. Food was removed 2 hours prior to dark onset. Shortly before dark onset, shrews (*n* = 9, ~59 ± 2 g) received intraperitoneal injection of semaglutide (10, 50, 100, and 500 nmol/kg) or vehicle. Shrews had ad libitum access to powdered food through a circular (3 cm in diameter) hole in the cage. Food intake was evaluated using our custom-made “feedometers,” consisting of a standard plexiglass rodent housing cage (29 cm by 19 cm by 12.7 cm) with mounted food hoppers resting on a plexiglass cup (to account for spillage). Food intake was manually measured at 6, 24, 48, and 72 hours postinjection. Body weight was taken at 0, 24, 48, and 72 hours. Treatments occurred in a within-subject, counterbalanced design and were 7 days apart.

To assess the emetogenic effects of semaglutide treatment, shrews (*n* = 9) were habituated to intraperitoneal injections and to clear plastic observation chambers (23.5 cm by 15.25 cm by 17.8 cm) for two consecutive days prior to experimentation. The animals were injected intraperitoneally with semaglutide (10, 50, 100, and 500 nmol/kg) or vehicle and then video recorded (Vixia HF-R62, Canon) for 120 min. After 120 min, the animals were returned to their cages. Treatment rounds were conducted within-subject and separated by 7 days. Analysis of emetic episodes was measured by a trained observer blinded to treatment groups. Emetic episodes were characterized by strong rhythmic abdominal contractions associated with either oral expulsion from the GI tract (i.e., vomiting) or without the passage of materials (i.e., retching). Latency to the first emetic episode, the total number of emetic episodes, and the number of emetic episodes per minute were quantified.

### Comparison of semaglutide, GIP-085, and GIP-085/semaglutide treatments on energy balance and emesis in shrews

First, we compared the effects of semaglutide (500 nmol/kg), GIP-085 (300 nmol/kg), GIP-085/semaglutide, or vehicle on food intake and body weight in the same animals (*n* = 8, ~54 ± 2 g) using in a within-subject, counterbalanced design. This experiment was conducted as described above. Each treatment was 7 days apart.

We then evaluated the emetogenic actions of semaglutide (500 nmol/kg), GIP-085 (300 nmol/kg), GIP-085/semaglutide, or vehicle in a different cohort of shrews (*n* = 10). The animals were habituated to the experimental conditions as described above, injected intraperitoneally with semaglutide (5000 nmol/kg), GIP-085 (300 nmol/kg), GIP-085/semaglutide, or vehicle and then video-recorded (Vixia HF-R62, Canon) for 120 min. After 120 min, the animals were returned to their cages. Treatments were separated by 7 days. Emesis parameters were quantified as described above.

### Dose-response effects of semaglutide on glycemic control in shrews

Similar to what was described above, each shrews received intraperitoneal injection of tirzepatide (10, 100, and 1000 nmol/kg) or vehicle (10 ml/kg body weight) followed 30 min later by a glucose bolus injection (2 g/kg, ip). Blood glucose readings were taken at −30, 0, 20, 40, 60, and 120 min post-glucose administration. This study was conducted using a partial within-subject design, with each animal receiving two treatments separated by a 7-day interval.

### Dose-response effects of tirzepatide on energy balance and emesis in shrews

First, we compared the effects of tirzepatide (10, 100, and 1000 nmol/kg) or vehicle on food intake and body weight in the same animals (*n* = 9, ~54 ± 3 g) using in a within-subject, counterbalanced design. This experiment was conducted as described above. Each treatment was 7 days apart.

We then evaluated the emetogenic actions of tirzepatide (10, 100, and 1000 nmol/kg) or vehicle in a different cohort of shrews (*n* = 9). The animals were habituated to the experimental conditions as described above. Treatments were separated by 7 days. Emetic episodes were recorded and quantified as described above.

### cAMP signaling assay

HEK Freestyle cells (Thermo Fisher Scientific, R79007) were seeded in suspension at a density of 250,000 cells/ml and then subsequently transfected with untagged rat or shrew GIPR constructs using Fugene-6 (Promega, E2691). After 48 hours, these cells were spun down and resuspended in assay buffer (Dulbecco’s modified Eagle’s medium, Gibco 31053) containing 0.1% casein. Cells were diluted to 1000 cells per well and added to 384-well white microplates (Costar, 3570) containing the indicated peptide ligands that were prepared via acoustic direct dilution in buffer containing 250 μM IBMX (total reaction volume of 20 μl), followed by incubation for 30 min at 37°C. Cells were subsequently lysed by the addition of 10 μl of d2-labeled cAMP competitor conjugate and 10 μl of cryptate-conjugated detection antibody (Revvity, 62AM4PEC) and incubated at room temperature for 1 hour. Quantification of time-resolved fluorescence resonance energy transfer was done using an Envision plate reader with transformation of ratios to nanomolar values using external synthetic cAMP standards contained in a plate that was processed in parallel, and normalized percent values were fit using GraphPad Prism and a four-parameter logistic model.

### Quantification and statistical analysis

Body weight changes were calculated by subtracting body weight on any given posttreatment day from the rat’s weight on the day of injection. In all behavioral studies, food intake, kaolin intake, and body weight changes data were analyzed using ordinary or repeated measures one-way or two-way analyses of variance (ANOVAs) followed by Tukey’s post hoc tests. An exception is the data shown in Fig. 3 (G and H), which were analyzed with a repeated measures mixed-effects models followed by Tukey’s post hoc tests. Emetic episodes were analyzed using repeated measures one-way ANOVAs followed by Tukey’s post hoc tests. Conditioned aversive reactions were analyzed with two-way ANOVA followed by Tukey’s post hoc tests. Glucose levels were analyzed using ordinary or repeated measures two-way ANOVA followed by Tukey’s post hoc tests. AUCs were calculated from 0 to 120 min using the trapezoidal method. Resulting AUCs were analyzed using ordinary or repeated measures one-way ANOVA followed by Tukey’s post hoc tests. All graphical data were expressed as means ± SEM. For all statistical tests, a *P* value of less than 0.05 was considered significant. Information on replicates and significance is reported in the figure legends. All data were analyzed using Prism 10 GraphPad Software (San Diego, CA). Power analyses were performed ahead according to a model of 20% minimum effect size (α = 0.05; β = 0.10) for kaolin/food intake, with estimated SD based on our published work.
