# Supplementary material for: Hypophagia and body weight loss by tirzepatide are accompanied by fewer GI adverse events compared to semaglutide in preclinical models
Source: Sci Adv. 2025 Jun 18;11(25):eadu1589. doi: 10.1126/sciadv.adu1589 (PMC12175907; doi:10.1126/sciadv.adu1589)
Supplement: Supplementary file 1 — Figs. S1 and S2 [file sciadv.adu1589_sm.pdf]

Science Advances
AAAS

Supplementary Materials for

Hypophagia and body weight loss by tirzepatide are accompanied by fewer GI adverse events compared to semaglutide in preclinical models

Tito Borner *et al.*

Corresponding author: Tito Borner, tborner@usc.edu; Matthew R. Hayes, hayesmr@pennmedicine.upenn.edu; Bart C. De Jonghe, bartd@nursing.upenn.edu

Sci. Adv. **11**, eadu1589 (2025)
DOI: 10.1126/sciadv.adu1589

This PDF file includes:

Figs. S1 and S2

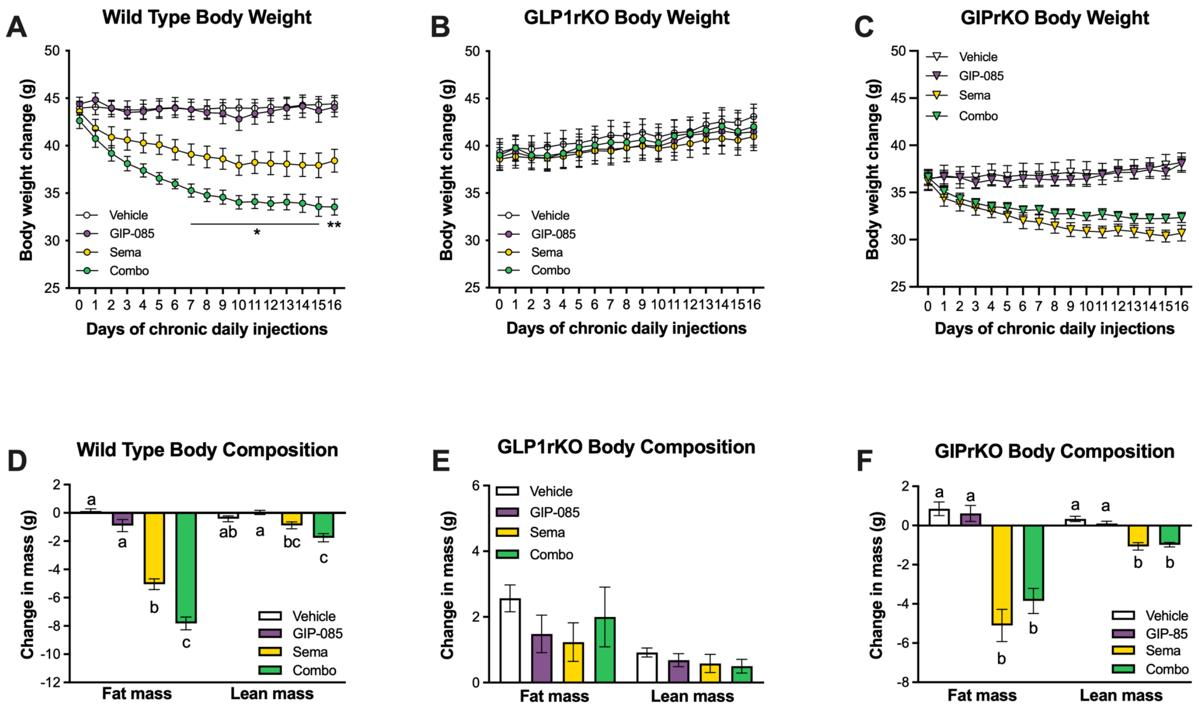

Supplementary Figure 1: Effects of semaglutide, GIP-085, and combo treatment on body weight and body composition in WT, GLP-1R KO and GIPR KO obese mice

A) Chronic systemic GIPR/GLP-1R dual agonism induces greater weight loss compared to semaglutide alone in diet-induced obese (DIO) wild-type mice (GIP-085: 300 nmol/kg; semaglutide: 3 nmol/kg, n=5-6 per group, * $P < 0.05$, ** $P < 0.01$).

B) The weight lowering effects of semaglutide and dual semaglutide/GIP-085 treatments are absent in DIO GLP-1R KO mice (GIP-085: 300 nmol/kg; semaglutide: 3 nmol/kg, n=6 per group).

C) No synergistic body weight lowering effects were observed in DIO GIPR KO mice following dual treatment (GIP-085: 300 nmol/kg; semaglutide: 3 nmol/kg, n=6 per group).

D) Effects of chronic treatments on fat and lean mass in DIO wild-type mice (GIP-085: 300 nmol/kg; semaglutide: 3 nmol/kg, n=5-6 per group). The total fat mass at the end of the experiment was Veh: 17.9±0.5g; GIP-085: 17.1±1.0g; Sema: 12.2±0.8g; Combo: 9.4±0.8g. The total lean mass at the end of the experiment was: Veh: 24.8±0.6g; GIP-085: 25.4±0.3g; Sema: 24.6±0.7g; Combo: 22.8±0.4g.

E) No changes in body composition occurred following chronic semaglutide and dual semaglutide/GIP-085 treatments in GLP-1R KO DIO mice (n=6 per group). The total fat mass at the end of the experiment was Veh: 17.3±0.8g; GIP-085: 15.9±1.2g; Sema: 15.5±0.9g; Combo:

16.6±1.5g. The total lean mass at the end of the experiment was: Veh: 24.1±0.6g; GIP-085: 24.0±0.4g; Sema: 23.9±0.7g; Combo: 23.7±0.6g.

F) Dual semaglutide/GIP-085 treatment did not lead to greater fat mass loss compared to semaglutide alone in DIO GIPR KO mice (n=6 per group). The total fat mass at the end of the experiment was Veh: 14.2±0.6g; GIP-085: 13.8±0.7g; Sema: 8.1±0.6; Combo: 9.9±0.6g. The total lean mass at the end of the experiment was: Veh: 22.3±0.5g; GIP-085: 22.3±0.4g; Sema: 20.9±0.3g; Combo: 21.0±0.3g.

All data expressed as mean ± SEM. Data in **(A, B, C)** were analyzed with repeated measures two-way ANOVA followed by Tukey's post hoc tests. Data in **(D, E, F)** were analyzed with one-way ANOVA followed by Tukey's post hoc tests. Means with different letters are significantly different from each other (P < 0.05).

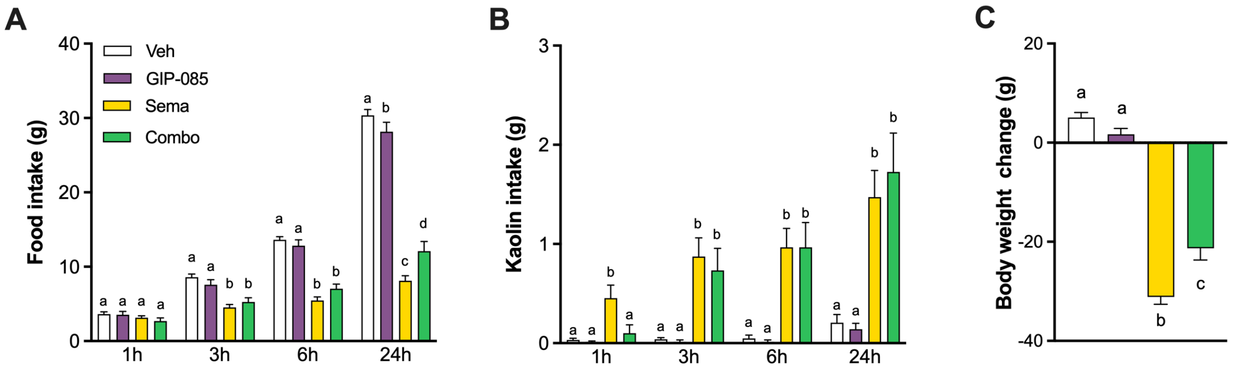

Supplementary Figure 2: GIPR agonism attenuates semaglutide-induced illness in rats.

A) Food intake following treatment with GIP-085 (300 nmol/kg), semaglutide (10 nmol/kg), combo or vehicle in rats. The anorectic effect of semaglutide was partially reduced by GIP-085 co-treatment (n=15 per group).

B) GIP-085 co-treatment attenuated acute kaolin intake induced by semaglutide (n=15 per group).

C) GIP-085 reduced semaglutide-induced body weight loss in rats (n=15 per group).

All data expressed as mean ± SEM. Data in **(A, B)** were analyzed with repeated measures two-way ANOVA followed by Tukey's post hoc tests. Data in **(C)** were analyzed with repeated measures one-way ANOVA followed by Tukey's post hoc tests. Means with different letters are significantly different from each other (P < 0.05).
